# Serum IgG levels to Epstein-Barr and measles viruses in patients with multiple sclerosis during natalizumab and interferon beta treatment

**DOI:** 10.1136/bmjno-2022-000271

**Published:** 2022-07-27

**Authors:** Linn Persson Berg, Marcus Eriksson, Sonia Longhi, Ingrid Kockum, Clemens Warnke, Elisabeth Thomsson, Malin Bäckström, Tomas Olsson, Anna Fogdell-Hahn, Tomas Bergström

**Affiliations:** 1 Department of Infectious Diseases, Institute of Biomedicine, University of Gothenburg, Gothenburg, Sweden; 2 Department of Clinical Microbiology, Sahlgrenska University Hospital, Gothenburg, Sweden; 3 Lab. Architecture et Fonction des Macromolécules Biologiques (AFMB), UMR 7257, Aix Marseille University and Centre National de la Recherche Scientifique (CNRS), Marseille, France; 4 Department of Clinical Neuroscience, Center for Molecular Medicine, Karolinska Institutet, Stockholm, Sweden; 5 Department of Neurology, Faculty of Medicine and University Hospital Cologne, University of Cologne, Cologne, Germany; 6 Mammalian Protein Expression Core Facility, Sahlgrenska Academy, University of Gothenburg, Gothenburg, Sweden

**Keywords:** multiple sclerosis, virology, medicine, immunology, infectious diseases

## Abstract

**Background:**

Patients with multiple sclerosis (MS) demonstrate higher seroprevalence of Epstein-Barr virus (EBV) and increased anti-EBV IgG levels in serum compared with healthy controls. Intrathecal antibody production to measles virus (MeV) is a common finding in patients with MS.

**Objective:**

To measure serum IgG reactivity to EBV glycoprotein 350 (gp350) and MeV nucleocapsid protein (N_CORE_) in patients with MS and healthy controls and to determine if reactivity changed in patients during interferon beta (IFNβ) and/or natalizumab (NAT) treatment. A secondary aim was to determine the seroprevalence of EBV in patients and controls.

**Methods:**

Patients with MS (n=728) were included from the Swedish pharmacovigilance study for NAT. Paired serum samples from 714 patients drawn before and during NAT treatment and paired samples from 170 patients during prior IFNβ treatment were analysed. In total, 156 patients were included in both groups. Samples from 144 matched blood donors served as controls. Indirect ELISA was applied using recombinant EBVgp350 and MeV N_CORE_ as antigens. EBVgp350 IgG seronegative samples were also analysed using EBV nuclear antigen 1 and viral capsid antigen (VCA).

**Results:**

Patients with MS showed higher serum levels of anti-EBVgp350 and anti-MeV N_CORE_ IgG compared with controls. During NAT treatment, the levels of anti-EBVgp350 and anti-MeV N_CORE_ IgG declined, compared with the relatively stable levels noted during prior IFNβ treatment. Ten patients failed to demonstrate anti-EBVgp350 IgG but did show detectable anti-VCA IgG, indicating EBV seropositivity. In contrast, 10/144 controls were EBV seronegative.

**Conclusions:**

Treatment with NAT, which is considered a selective immunosuppressive agent with a compartmentalised effect on the central nervous system, appeared to be associated with a moderate decrease in circulating IgG levels to EBVgp350 and MeV N_CORE_. All patients with MS were EBV IgG seropositive, supporting the potential role of EBV in the pathogenesis of MS.

**WHAT IS ALREADY KNOWN ON THIS TOPIC**

- Epstein-Barr virus (EBV) is serologically associated with multiple sclerosis (MS). Intrathecal antibody production to measles virus (MeV) is a common finding in patients with MS.

**WHAT THIS STUDY ADDS**

- Patients with MS showed increased IgG serum levels to EBV glycoprotein 350 (EBVgp350) and MeV nucleocapsid antigen (MeV N_CORE_) compared with healthy blood donors serving as controls. In patients with MS, serum levels of anti-EBVgp350 and anti-MeV N_CORE_ IgG decreased during treatment with natalizumab (NAT), whereas levels were relatively stable during previous interferon beta treatment. All 728 patients with MS were EBV IgG seropositive, while 10/144 of the controls were seronegative.

**HOW THIS STUDY MIGHT AFFECT RESEARCH, PRACTICE OR POLICY**

- NAT treatment may be associated with a decrease in anti-EBVgp350 and anti-MeV N_CORE_ IgG serum levels in patients with MS and the potential clinical significance requires further investigation. EBV’s potential role in the pathogenesis of MS is supported in the study as all 728 patients with MS were EBV seropositive.

## Introduction

Patients with multiple sclerosis (MS) display an increased IgG response to certain, but not all, neurotropic viruses compared with healthy controls.^1–8^ The increased intrathecal IgG response to measles virus (MeV), rubella virus and varicella-zoster virus (VZV), termed the MRZ reaction, is a characteristic finding and may serve as a supportive diagnostic test for MS.^4–6^ The MRZ reaction is due to increased IgG reactivity in the central nervous system (CNS). A few studies have also showed that patients with MS demonstrate increased serum anti-MeV IgG levels in response to both natural infection and vaccination.^3 4^ Moreover, it is established that the seroprevalence of Epstein-Barr virus (EBV) is higher in patients with MS,^9–11^ with increased serum anti-EBV IgG levels compared with healthy controls.^1 2^ Finally, The risk of developing MS increases following EBV seroconversion,^12^ and also following symptomatic EBV infection in the form of infectious mononucleosis.^10 13^ In contrast, previous studies have revealed a negative or no association between MS and cytomegalovirus (CMV) seropositivity.^7 8 12^

The reason underlying the increased IgG response to EBV and MeV in patients with MS remains unknown. The abnormal IgG response has been studied as a potential surrogate biomarker,^2 14–19^ but whether MS disease activity and treatment affects this serological landscape, and if so, how, remains largely unknown. One study showed a correlation between MeV IgG antibody index and MS disease activity.^14^ The possible correlation between EBV serology and disease activity in MS has been studied more extensively, but with contradictory results.^2 15 16^

At present, there is no cure for MS, but several disease-modifying therapies are available. Many patients with relapsing–remitting MS were previously treated with interferon beta (IFNβ). Among the growing arsenal of treatment regimens with improved efficacy, one of the earliest strikingly more effective immune-modulating therapies to receive approval was natalizumab (NAT, Tysabri),^20^ a recombinant, humanised monoclonal IgG_4_ antibody that inhibits leucocyte migration across the blood–brain barrier.^20^

Patients with MS are at higher risk of contracting certain infectious diseases, compared with the general population, and use of disease-modifying treatment may increase this risk.^21 22^ The risk for progressive multifocal leukoencephalopathy (PML) is increased during NAT treatment.^23^ Primary central nervous system lymphoma (PCNSL) and herpesvirus infections of the CNS have also been associated with NAT therapy.^24 25^ In our preceding study, we demonstrated that IgG reactivity to JC polyomavirus (JCV) and VZV declines in patients with MS during NAT treatment, but not during IFNβ therapy.^26^ In contrast, that study revealed a slight increase in IgG reactivity to CMV during treatment.^26^ To further investigate whether treatment affects the increased IgG response to MS-associated viruses in these patients, we aimed to assess the effects of IFNβ and NAT therapy on IgG reactivity to EBV and MeV.

Earlier research examining EBV seroreactivity in patients with MS has frequently been based on assays using viral capsid antigen (VCA) and/or early antigen in addition to the predominant Epstein-Barr virus nuclear antigen 1 (EBNA1).^1 2 10 11 15–19^ Previous studies have not found any change in serum anti-EBNA1 IgG levels during NAT therapy.^17–19^ One study did report an increase in serum anti-VCA IgG levels during NAT treatment,^18^ while another did not.^19^ To extend the knowledge about such IgG antibody reactivity during treatment of patients with MS, the present study analyses the IgG response to EBV glycoprotein 350 (EBVgp350), a major viral envelope protein previously not investigated in this context.

EBVgp350 has the potential to induce potent and specific IgG responses as demonstrated with some other herpesvirus glycoproteins, for example, VZV glycoprotein E.^27^ EBVgp350 is the most abundant envelope glycoprotein present on EBV particles^28^ and the main target for neutralising antibodies.^29^ Moreover, the generation of anti-EBVgp350 neutralising antibodies is associated with the EBV viral load in blood.^30^ The antibody response to viral envelope glycoproteins such as EBVgp350 and intranuclear antigens such as EBNA1 can show different kinetics and it is thus interesting to assay reactivities to both these antigens in patients with MS. Moreover, the link between using EBVgp350 as a serological antigen and the previous use of this protein in an EBV vaccine^31^ is intriguing in light of recent epidemiological associations between this virus and MS.^12^

Our research group has previously used the MeV nucleocapsid protein (N_CORE_) as a serological antigen to determine the specificity of anti-MeV IgG reactivity in patients with MS, their siblings and healthy controls.^4^ The EBVgp350 and MeV N_CORE_ antigens are based on single, immunogenic viral proteins and developed to be devoid of human/primate cellular remnants.^4 32^ Use of these types of antigens helps to minimise risk of detecting cross-reactive antibodies against viruses with similar epitopes and autoantibodies against cellular components, both of which may create false positive reactions in patients with autoimmune diseases such as MS. The aim of this study was to use highly specific serological assays to measure serum IgG reactivity to EBVgp350 and MeV N_CORE_ in patients with MS and healthy controls and to determine if serum IgG reactivity changes in patients treated with IFNβ and/or NAT. A secondary aim was to determine the seroprevalence of EBV in patients and controls.

## Methods

### Patients and controls

The serum samples analysed for this study were obtained from patients with MS enrolled in the Swedish pharmacovigilance study for NAT (IMSE).^33 34^ The initial cohort consisted of 1157 patients, all treated with NAT before March 2010.^26 33 34^ Our preceding study analysed samples from 844 patients after excluding 313 patients because of prior treatment with intravenous immunoglobulin or insufficient quantification of anti-JCV antibodies.^26^ There were 714 patients with sufficient serum left over for analysis, on which anti-viral IgG tests were performed for the purposes of the present study. Before initiation of NAT therapy, 115 patients were treatment naïve; the others were treated with IFNβ (n=396), Copaxone (n=101), Metoxantrone (n=32), Solu-Medrol (n=10) or different smaller regimes (n=27). Information on previous treatment was not available for 33 patients.

The samples from patients in the NAT group included one sample taken immediately prior to the first infusion of NAT, at time point 3 (t3) and the last available sample during NAT treatment, at time point 4 (t4). Median time between sampling was 12 months with IQR of 7–24 months. Additional serum samples from 170 patients in the initial NAT cohort had been obtained earlier during IFNβ treatment at time points 1 (t1) and 2 (t2) but 14 of these 170 patients lacked material from the samples obtained at t3 and t4, so only 156 patients were included in both the IFNβ and NAT groups. Median time between sampling in the IFNβ subgroup was 13 months with IQR of 7–25 months. Median time between t2 and t3 was 9 months, with IQR of 4–19 months. In total, 728 patients with MS were included in the present study and 144 age-matched and sex-matched blood donors served as controls. The online supplemental efigure S1 illustrates the patient material.

10.1136/bmjno-2022-000271.supp1 Supplementary data

### ELISA

The serum samples were analysed by ELISA for detection of IgG against two purified recombinant antigens, EBVgp350 and MeV N_CORE_, for which production and serological evaluations were previously described.^4 32^ The antigens were diluted to 1 µg/mL or 0.2 µg/mL, respectively, using 0.05 M carbonate buffer, pH 9.6. The diluted antigens were added to Nunc MaxiSorp 96-well ELISA microplates (Thermo Fisher Scientific, Roskilde, Denmark) and stored at 4°C for at least one night. Before use, the plates were washed three times with phosphate buffered saline (PBS) solution containing 0.05% Tween 20. A blocking solution, 2% non-fat dry milk/PBS, was added to the wells to avoid non-specific binding. The plates were incubated at room temperature for 30 min.

For analysis of both anti-EBVgp350 and anti-MeV IgG, the serum samples were diluted 1/400 in PBS containing 1% non-fat dry milk and 0.05% Tween 20. A few of the paired samples needed to be further diluted in a second session due to very high levels of anti-EBV IgG, which yielded too high optical density (OD) values. In total, paired samples from 22 patients in the NAT group, 14 in the IFNβ subgroup and 4 blood donors required further dilution to 1/1600 for anti-EBVgp350 IgG analysis. Paired samples (t1 and t2; t3 and t4) were always assayed next to each other in duplicate along with positive and negative controls in quadruplicate on the same microplate in the same session.

The plates were incubated at 37°C for 90 min and then rinsed three times with PBS solution containing 0.05% Tween 20. A secondary conjugated antibody, Alkaline Phosphatase AffiniPure F(ab')₂ Fragment Goat Anti-Human IgG (H+L) (Jackson ImmunoResearch Europe, Cambridgeshire, UK) was diluted 1/1000 in PBS containing 1% non-fat dry milk/PBS and 0.05% Tween 20 and added to the wells. The plates were incubated for 60 min at 37°C and then washed six times with PBS solution containing 0.05% Tween 20. Next the substrate solution, phosphatase substrate (Phosphatase Substrate, Sigma-Aldrich, St. Louis, USA) was dissolved and diluted to 1 mg/mL in diethanolamine buffer pH 9.8, then added to the plates. The plates were shaken in the Thermo Scientific Multiskan FC spectrophotometer before measuring the OD of the colour reaction. The wavelength of the main filter was 405 nm and the reference filter 620 nm. Based on previous studies, the seropositivity cut-off for the EBVgp350 ELISA was set to an OD of 0.162^32^ and the cut-off for the MeV N_CORE_ ELISA was set to the mean absorbance value for the negative control +0.2 absorbance units.^4^ The intra-assay and interassay coefficient of variation for the EBVgp350 ELISA was 4.2% and 12%, respectively, and for the MeV N_CORE_ ELISA the corresponding variances were 4.3% and 10%. The EBVgp350 IgG seronegative samples were analysed by the ALINITY i immunoassay system (Abbott, Abbott Park, Illionois, USA) using EBNA1 and VCA as antigens (Abbott, Scandinavia AB).

### Delta OD values

To explore eventual changes in anti-EBVgp350 and anti-MeV N_CORE_ IgG levels in the paired serum samples obtained during IFNβ treatment (n=170) at t1 and t2 and before (t3) and during (t4) NAT treatment (n=714), the OD value of the first sample taken at t1 or t3 was subtracted from the OD value of the second sample taken at t2 or t4 (ie, t2 minus t1; t4 minus t3), thereby yielding delta (∆) OD values. ∆OD values above zero indicate an increased level and values below zero indicate a decreased level of anti-EBVgp350 or anti-MeV N_CORE_ IgG.

### Total serum IgG levels

Fifty patients who had paired samples taken both during IFNβ treatment (t1 and t2) and before (t3) and during NAT treatment (t4), were randomly selected for analysis of total serum IgG levels. The samples belonging to the same patient were analysed in the same session on the same plate by Human IgG immunoperoxidase assay to determine IgG in human samples (Immunology Consultants Laboratory, Portland, Oregon, USA) according to the manufacturer’s instructions.

### Statistical methods

All statistical analyses were carried out using SPSS Statistics V.27. The Mann-Whitney U test was used to compare anti-EBVgp350 and anti-MeV N_CORE_ IgG levels in patients with MS during IFNβ treatment at t1 and in the blood donor controls. The changes of anti-EBVgp350 and anti-MeV N_CORE_ IgG levels, between the samples collected during IFNβ treatment at t1 and t2 and before (t3) and during (t4) NAT treatment, were analysed using the Wilcoxon signed-rank test. The Bonferroni correction was used because multiple statistical tests were performed in the study. All statistical tests were two sided and p values<0.008 (0.05/6 due to Bonferroni correction) were considered statistically significant.

For exploratory purposes, the Pearson correlation coefficient was used to investigate correlation between treatment time with NAT and change in IgG levels. The time between t3 and t4 measured in months was correlated to anti-EBVgp350 and anti-N_CORE_ IgG ∆OD values. Wilcoxon signed-rank test was used to compare the total IgG levels between the paired samples.

## Results

### Anti-EBVgp350 and anti-MeV N_CORE_ IgG levels in patients with MS and controls

**Figure 1:**
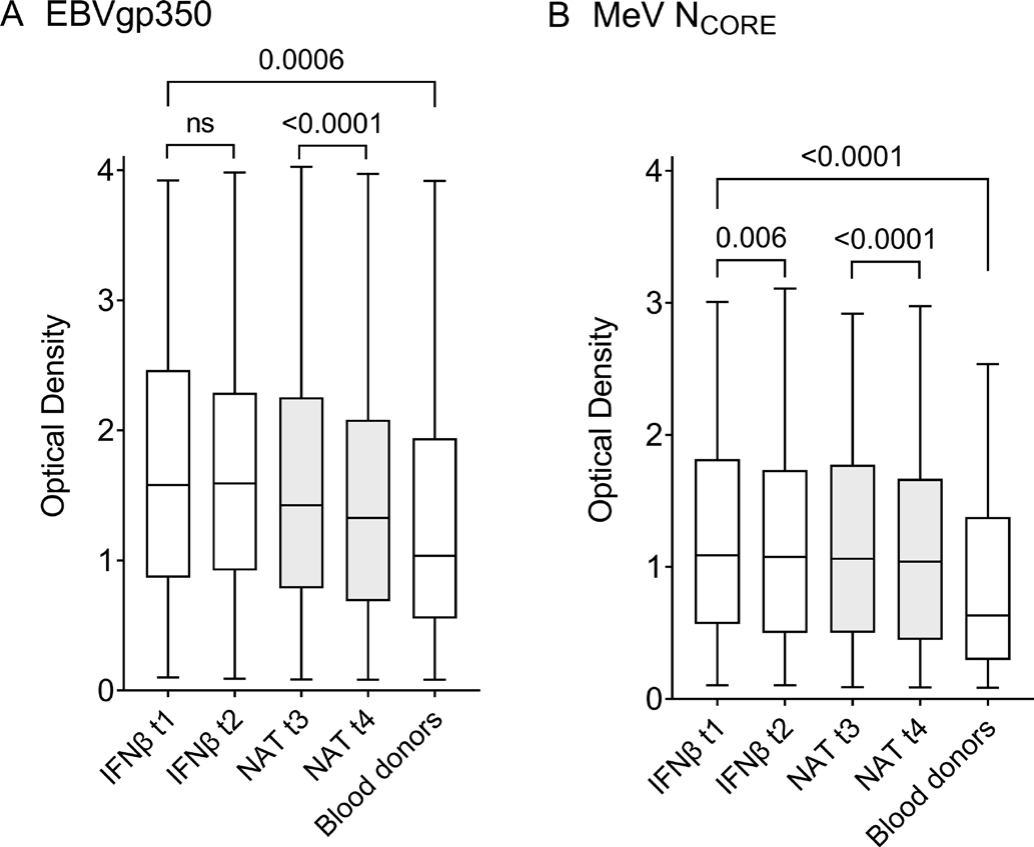
IgG reactivity measured as optical density in serum samples against (A) Epstein-Barr virus glycoprotein 350 (EBVgp350) and (B) measles virus nucleocapsid protein (MeV N_CORE_). There were 170 patients with multiple sclerosis in the interferon beta (IFNβ) subgroup sampled at time point 1 (t1) and t2, 714 patients in the natalizumab (NAT) group, sampled at t3 and t4 and 144 blood donors. The boxplots demonstrate minimum, quartile 1, median, quartile 3 and maximum. The Mann-Whitney U test was used to compare the IgG levels in patients during IFNβ treatment at t1 and blood donors. The Wilcoxon signed-rank test was used to compare the anti-EBVgp350 and anti-MeV N_CORE_ IgG levels between the samples collected during IFNβ treatment at t1 and t2 and before (t3) and during (t4) NAT therapy. P values<0.008 (0.05/6 due to Bonferroni correction) were considered statistically significant.

Sex and age distribution were similar among patients with MS and the control group of 144 healthy blood donors (table 1). In the statistical analysis, blood donor antibody levels were compared with those of patients with MS during IFNβ treatment at t1, since the patients were most treatment naïve at this point. Patients with MS demonstrated higher levels of both anti-EBVgp350 (p=0.0006) and anti-MeV N_CORE_ IgG (p<0.0001) (figure 1 and table 2).

**Table 1:** Patient characteristics

	Total n	Female	Male	Median age (range)	Median ∆t months (range)
IFNβ subgroup t1–t2	170	106 (62%)	64 (38%)	37 (16–58)	13 (1–59)
NAT group t3–t4	714	500 (70%)	214 (30%)	37 (12–63)	12 (1–38)
Blood donors	144	100 (69%)	44 (31%)	35 (18–63)	

The analysed serum samples were obtained from patients with multiple sclerosis enrolled in the Swedish pharmacovigilance study for natalizumab (NAT). The interferon beta (IFNβ) subgroup is a subgroup of the NAT group defined by samples available prior to NAT therapy at time point 1 (t1) and t2. The NAT group consists of samples taken before NAT treatment, at t3 and during NAT treatment, at t4. Sex-matched and age-matched blood donors were included as a control group. Δt is duration of time in months between samples taken at t1–t2 and t3–t4.

**Table 2:** IgG antibodies to EBVgp350 and MeV N_CORE_ in patients and controls

	Anti-EBVgp350 IgG		Anti-MeV N_CORE_ IgG	
	Median	IQR	Median	IQR
IFNβ t1 (n=170)	1.58	0.869–2.46	1.09	0.566–1.82
IFNβ t2 (n=170)	1.59	0.922–2.29	1.08	0.501–1.73
NAT t3 (n=714)	1.42	0.786–2.25	1.06	0.501–1.77
NAT t4 (n=714)	1.33	0.688–2.08	1.04	0.448–1.67
Blood donors (n=144)	1.04	0.554–1.94	0.632	0.296–1.38

Anti-Epstein-Barr virus glycoprotein 350 (EBVgp350) and anti-measles virus nucleocapsid (MeV N_CORE_) IgG antibodies were measured as optical density in serum samples. There were 170 patients with multiple sclerosis in the interferon beta (IFNβ) subgroup, sampled at time point 1 (t1) and t2, 714 patients in the natalizumab (NAT) group, sampled at t3 and t4 and 144 blood donors in the control group. The table shows median with IQR for each group of samples.

### Anti-EBVgp350 and anti-MeV N_CORE_ IgG levels during IFNβ and NAT treatment

**Figure 2:**
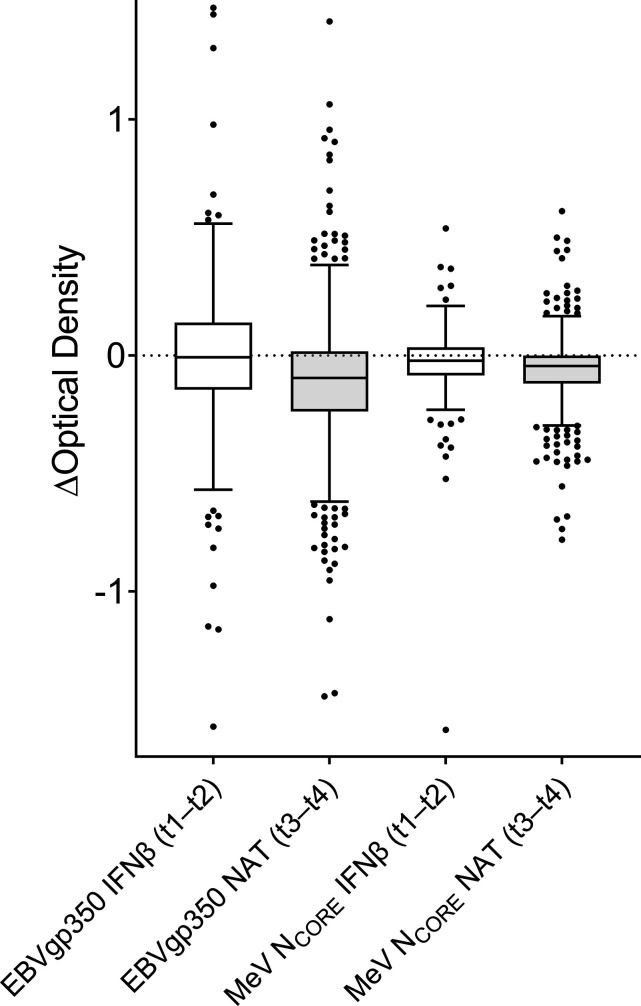
Tukey box plot illustrating changes in anti-Epstein-Barr virus glycoprotein 350 (EBVgp350) and anti-measles virus nucleocapsid (MeV N_CORE_) IgG reactivity in serum samples from patients with multiple sclerosis. In all, 170 patients were sampled during interferon (IFNβ) treatment at time point 1 (t1) and t2 and 714 patients were sampled before (t3) and during natalizumab (NAT) treatment (t4). The relative levels of anti-EBVgp350 and anti-MeV N_CORE_ IgG were analysed by indirect ELISA and measured as optical density (OD). The OD value for the first sample taken at t1 or t3 was subtracted from the second value at t2 or t4, creating delta (∆) OD values.

Changes in IgG levels between the paired samples collected during IFNβ treatment at t1 and t2 (n=170) and before (t3) and during (t4) NAT treatment (n=714) are shown as total OD values in figure 1 and table 2 and as ∆OD in figure 2. Anti-EBVgp350 IgG levels in the IFNβ subgroup did not change between t1 and t2; only 93/170 (55%) patients had lower levels in the t2 follow-up sample compared with the initial sample at t1. In contrast, the NAT group demonstrated a decline in anti-EBVgp350 IgG between t3 and t4 (p<0.0001). In all, 509/714 (71%) paired samples demonstrated lower anti-EBVgp350 IgG levels at t4 follow-up compared with samples drawn at t3.

The change in anti-MeV N_CORE_ IgG levels between t1 and t2 in the IFNβ-treated subgroup was marginal, though there was a decline (p=0.006). In all, 95/170 (56%) paired samples demonstrated a decline in anti-MeV N_CORE_ IgG levels in the follow-up sample at t2 compared with the initial sample at t1. In the NAT group, there was a more pronounced decline in anti-MeV N_CORE_ IgG levels between t3 and t4 (p<0.0001) where 538/714 (75%) demonstrated lower anti-MeV N_CORE_ IgG levels in the follow-up sample at t4 than at t3.

A weak negative correlation was observed between treatment time with NAT and magnitude of change in anti-EBVgp350 and anti-MeV N_CORE_ IgG levels between samples collected at t3 and t4, where the correlation coefficient for anti-EBVgp350 IgG was −0.087 (p=0.021) and for anti-MeV N_CORE_ IgG was −0.083 (p=0.027).

### Seroprevalence

In the IFNβ subgroup, 3/170 (1.8%) patients were EBVgp350 IgG seronegative at both t1 and t2. Two of these patients were also seronegative at t3 and t4, while the third patient became seropositive. In the NAT group, 11/714 (1.5%) patients were seronegative at both t3 and t4. One of these patients was seropositive during prior IFNβ treatment but showed low anti-EBVgp350 IgG levels. Three additional patients in the NAT group demonstrated low anti-EBVgp350 IgG levels at t3 and became seronegative at t4. Thus, 14/714 samples were seronegative at t4. As described above, two seronegative patients in the NAT group were also seronegative during prior IFNβ treatment. In total, 10 patients had no sample demonstrating EBVgp350 IgG reactivity. In the blood donor control group, 14/144 (9.7%) were seronegative. Analysis of EBVgp350 IgG seronegative samples using VCA and EBNA1 as antigens demonstrated that all patients with MS were VCA IgG seropositive. One patient with MS was EBNA1 IgG seronegative, while another had anti-EBNA1 IgG in the grey (borderline) zone. In contrast, 10 blood donors were both EBNA1 and VCA IgG seronegative. To summarise, 10/144 (6.9%) blood donors were EBV seronegative but all 728 patients with MS were EBV IgG seropositive.

Seroprevalence of anti-MeV IgG was lower than seroprevalence of anti-EBV in both patients with MS and blood donors. In the IFNβ subgroup, 15/170 (8.8%) samples were anti-MeV N_CORE_ IgG seronegative at t1 and 18/170 (11.%) at t2. In the NAT group, 80/714 (11%) samples were seronegative at t3 and 108/714 (15%) at t4. Among the blood donors 41/144 (28%) were anti-MeV N_CORE_ IgG seronegative.

### Total serum IgG levels

The total serum IgG levels in the 50 patients in the IFNβ subgroup showed a decrease (p<0.0001) between samples drawn at t1 (median 9.77 g/L, IQR 8.05–11.3) and at t2 (median 8.06 g/L, IQR 6.77–9.53). For the same patients, there was no change in total IgG levels between samples obtained before NAT therapy at t3 (median 10.3 g/L, IQR 8.40–12.3) and samples obtained during NAT therapy at t4 (median 10.7 g/L, IQR 8.98–11.8).

## Discussion

This study addresses how different treatment regimens for patients with MS may alter serum IgG responses to two MS-associated viruses, EBV and MeV. Our findings here, along with the results from a recent paper from our group,^35^ indicate that patients with MS have higher anti-EBVgp350 IgG levels compared with healthy controls. Within this context, it is interesting to note that anti-EBVgp350 IgG levels remain increased in patients with mononucleosis at 6 months^30^ and even at 10-year follow-up.^35^ The finding that anti-MeV N_CORE_ IgG levels are higher among patients with MS than among controls is consistent with previous research.^3 4^

In patients with MS, levels of anti-EBVgp350 and anti-MeV N_CORE_ IgG decreased during NAT treatment. The decline in anti-MeV N_CORE_ IgG seemed less pronounced, but a direct comparison using different serological assays may be questioned. There was no change in anti-EBVgp350 IgG levels during IFNβ therapy. The decline in MeV N_CORE_ IgG levels during IFNβ therapy was small and may not be convincing in this context since only around half of the patients had lower levels in the t2 follow-up sample compared with the initial t1 sample. The knowledge that the IgG antibody response to EBV is usually lifelong, without showing any significant decline over time,^36^ and that the anti-MeV IgG response in patients with MS tends to increase over time in response to both natural infection and vaccination,^3^ suggest that the decreased antibody levels may be associated with NAT therapy. The weak correlation between treatment duration of NAT and decline of anti-EBVgp350 and anti-MeV N_CORE_ IgG levels may increase with longer duration of treatment.

Our preceding study showed an altered IgG response to JCV and two herpesviruses, VZV and CMV, during NAT therapy: anti-JCV and anti-VZV IgG levels declined, whereas anti-CMV IgG increased slightly.^26^ Whereas, the decline in anti-JCV antibodies might be directly linked to the increased risk for PML, the decrement of antibodies against the other viruses have so far not been associated with PCNSL or severe herpesvirus infections of the CNS but the matter has not been thoroughly investigated. The lack of a demonstrable decline in serum anti-EBNA1 and/or anti-VCA IgG in previous studies of NAT treatment^17–19^ could be due to differences in sensitivity of viral antigens or biological purposes for these antibodies compared with the neutralising function of the antibodies to EBVgp350, or possibly to the small sample sizes in some of these studies. Moreover, prior studies, using considerably smaller sample sizes than the current study, showed no decline in serum anti-MeV IgG levels during NAT treatment.^6 37^ Some studies have demonstrated a decline in total serum IgG levels during NAT treatment,^37–39^ but one study was only able to show a small IgG reduction in the longitudinal portion,^37^ while another failed to demonstrate a decline altogether.^6^ The decline in anti-EBVgp350 and anti-MeV N_CORE_ IgG could possibly reflect an overall decrease in IgG levels. However, we could in a randomly selected population of patient samples assayed for total IgG only find a decline during IFNβ treatment but no decline at all during NAT therapy. The slight increase in anti-CMV IgG levels during NAT therapy observed in our previous study^26^ suggests that NAT treatment does not suppress antibody production against all viruses. NAT therapy may alter antibody responses through immunosuppressive effects^26 38 40^ or possibly more indirectly by decreased inflammation-driven antigen exposure of those viral antigens to which patients with MS tend to over-react to.

The few samples that were EBVgp350 IgG seronegative had detectable antibodies to EBNA1 and/or VCA, indicating EBV seropositivity in all 728 patients with MS. EBV infection is suggested as a prerequisite for developing MS^9 12^ and large-scale EBV seroprevalence studies such as this one are therefore important. The use of several immunoassays increases the ability to identify EBV seropositivity^9 11^ and our EBVgp350 ELISA can be used as a complement to other routinely used methods. EBV seronegative patients with suspected clinically isolated syndrome/MS are rare, but in those cases, such EBV seronegativity may be a useful biomarker to identify patients who warrant further investigation.

NAT therapy was not associated with a decrease in anti-EBVgp350 and/or anti-MeV N_CORE_ IgG levels in approximately one fourth of patients with MS, which may be one caveat to the current study. A longer follow-up period would be of interest but may pose some hazard since the risk of PML increases with duration of NAT treatment.^23^ Another limitation is that the groups were not completely matched in the present study since 14 patients in the IFNβ subgroup only had sufficient serum material left from samples obtained during IFNβ therapy but not before and during NAT therapy. Our study focused solely on serum samples, why further investigation to correlate serum anti-EBVgp350 and anti-MeV N_CORE_ IgG levels to paired cerebrospinal fluid samples would be warranted. In addition, it would be of interest to conduct similar studies in other parts of the world to investigate if the findings in this study can be repeated in different populations.

## Conclusion

Before initiation of NAT treatment, patients with MS demonstrated higher serum anti-EBVgp350 and anti-MeV N_CORE_ IgG levels compared with controls. The elevated anti-EBVgp350 and anti-MeV N_CORE_ IgG levels in patients with MS declined during NAT treatment, though they had remained relatively stable during prior IFNβ therapy. The potential clinical significance of these findings requires further investigation, which may include studies related to a therapeutic effect of NAT against an inflammatory response against these viruses in MS. All 728 patients with MS were EBV IgG seropositive supporting a previously suggested potential role of EBV in the pathogenesis of MS.

## Data Availability

Data are available upon reasonable request.
