# Supplementary material for: Serum IgG levels to Epstein-Barr and measles viruses in patients with multiple sclerosis during natalizumab and interferon beta treatment
Source: BMJ Neurol Open. 2022 Jul 27;4(2):e000271. doi: 10.1136/bmjno-2022-000271 (PMC9335035; doi:10.1136/bmjno-2022-000271)
Supplement: Supplementary data [file bmjno-2022-000271supp001.pdf]

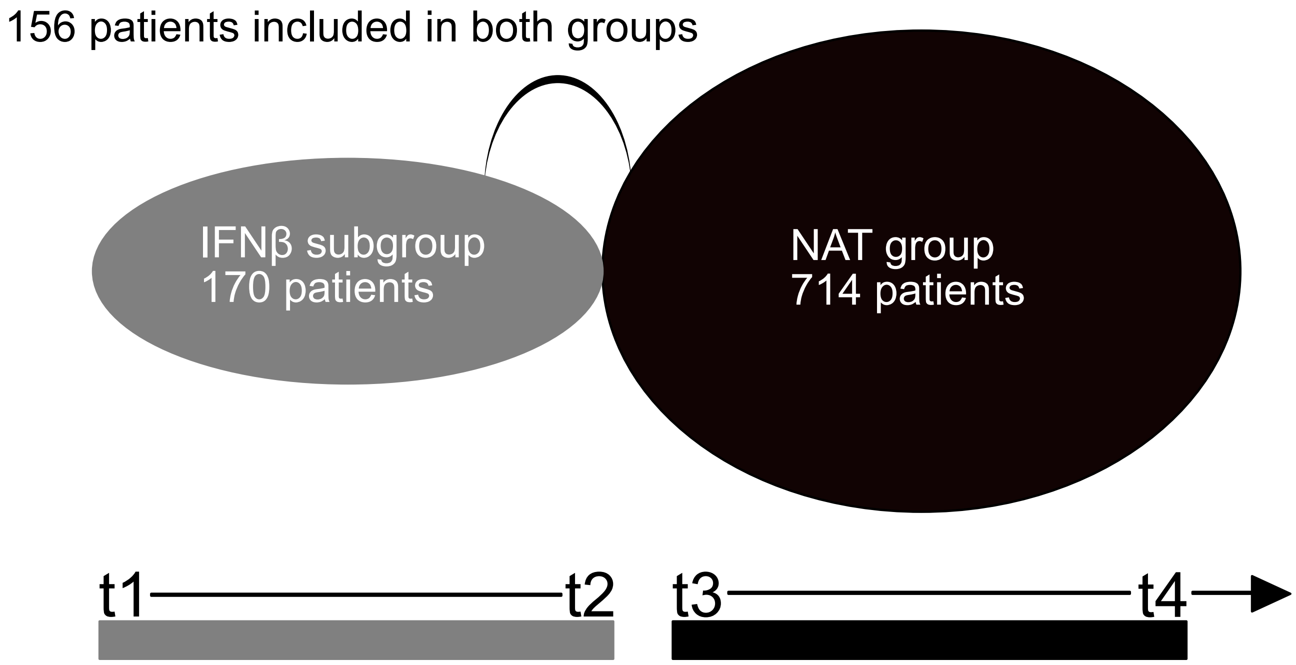

Efigure S1

In all, 170 patients with multiple sclerosis were sampled during interferon beta (IFNβ) treatment at time point 1 (t1) and t2. Median time between sampling was 13 months. The 170 patients in the IFNβ subgroup were subsequently treated with natalizumab (NAT). The NAT group consisted of 156 of these patients and 558 additional patients with MS initiating NAT therapy (n=714). The paired serum samples from the 714 patients in the NAT group were obtained immediately prior to the first infusion of NAT, at time point 3 (t3) and the last available sample during NAT treatment, at time point 4 (t4). Median time between sampling was 12 months.
